# *Sp*Bark Suppresses Bacterial Infection by Mediating Hemocyte Phagocytosis in an Invertebrate Model, *Scylla paramamosain*

**DOI:** 10.3389/fimmu.2019.01992

**Published:** 2019-08-23

**Authors:** Xin-Cang Li, Jian Zhou, Jun-Fang Zhou, Yue Wang, Hongyu Ma, Yuan Wang, Shu Zhao, Wen-Hong Fang

**Affiliations:** ^1^Key Laboratory of East China Sea Fishery Resources Exploitation, Ministry of Agriculture, East China Sea Fisheries Research Institute, Chinese Academy of Fishery Sciences, Shanghai, China; ^2^Guangdong Provincial Key Laboratory of Marine Biotechnology, Shantou University, Shantou, China

**Keywords:** scavenger receptor-like protein, C-type lectin domain, LPS-binding activity, bacterial clearance, phagocytosis activity, *Scylla paramamosain*

## Abstract

Scavenger receptors are cell surface membrane-bound receptors that typically bind multiple ligands and promote the removal of endogenous proteins and pathogens. In this study, we characterized a novel scavenger receptor-like protein, namely, *Sp*Bark. *SpBark* was upregulated in hemocytes after challenges with bacteria, suggesting that it might be involved in antibacterial defense. *Sp*Bark is a type I transmembrane protein with four extracellular domains, including three scavenger receptor cysteine-rich domains (SRCRDs) and a C-type lectin domain (CTLD). Western blot assay showed that *Sp*Bark CTLD possessed a much stronger binding activity to tested microbes than the three SRCRDs. It also exhibited apparent binding activities to lipopolysaccharide (LPS) and acetylated low-density lipoprotein (ac-LDL), whereas the other SRCRDs showed much lower or no binding activities to these components. Agglutination activities were observed in the presence of Ca^2+^ by incubating microorganisms with *Sp*Bark CTLD instead of SRCRDs. These results suggested that *Sp*Bark CTLD was the major binding site for ac-LDL and LPS. Coating *Vibrio parahemolyticus* with *Sp*Bark CTLD promoted bacterial clearance *in vivo*. This finding indicated that *Sp*Bark might participate in the immune defenses against Gram-negative bacteria through a certain mechanism. The promotion of bacterial clearance by *Sp*Bark was further determined using *SpBark*-silenced crabs injected with *V. parahemolyticus*. *SpBark* knockdown by injection of *SpBark* dsRNA remarkably suppressed the clearance of bacteria in hemolymph. Meanwhile, it also severely restrained the phagocytosis of bacteria. This finding suggested that *SpBark* could modulate the phagocytosis of bacteria, and the promotion of bacterial clearance by *Sp*Bark was closely related to *Sp*Bark-mediated phagocytosis activity. The likely mechanism of bacterial clearance mediated by *Sp*Bark was as follows: *Sp*Bark acted as a pattern recognition receptor, which could sense and bind to LPS on the surface of invading bacteria with its CTLD in hemolymph. The binding to LPS made the bacteria adhere to the surface of hemocytes. This process would facilitate phagocytosis of the bacteria, resulting in their removal. This study provided new insights into the hemocyte phagocytosis mechanisms of invertebrates and the multiple biological functions of Bark proteins.

## Introduction

Due to the lack of adaptive immunity, invertebrates rely on innate immunity to resist microbial invasion (1). Pattern recognition receptors (PRRs) participate in host immune defense by binding to specific ligands, resulting in the occurrence of innate immune responses (2, 3). These PRRs comprise structurally diverse domains that can recognize different pathogens by sensing specific pathogen-associated molecular patterns, including microbial polysaccharides, glycolipids, lipoproteins, nucleotides, and nucleic acids. To date, various PRRs, such as C-type lectins, Toll-like receptors, and scavenger receptors, have been identified in many invertebrates and vertebrate species (4–7).

Scavenger receptors are cell surface receptors that typically bind multiple ligands and promote the removal of non-self or altered-self targets (8). These proteins constitute a “superfamily” of membrane-bound receptors that were initially thought to bind and internalize modified low-density lipoprotein (mLDL). They were later found to play a role in binding to various ligands, including endogenous proteins and pathogens (9, 10). Scavenger receptors are currently proposed to be categorized into 12 classes on the basis of their structural diversities and biological functions (11). Each member in different classes exhibits a similar structure, including the intracellular portion, transmembrane domain, and extracellular region (10). The extracellular region always contains one or more functional domains, such as collagenous domain, scavenger receptor cysteine-rich domain (SRCRD), and C-type lectin domain (CTLD), thereby exhibiting a wide range of biological functions.

SRCRDs are present in a variety of host defense-related proteins, including several classes of scavenger receptors (11–14). They are ~100–110 amino acids long with 6–8 conserved cysteines predicted to form 3–4 disulfide bonds. SRCRD-containing proteins can bind a variety of ligands, including mLDL, structure proteins of certain viruses, bacteria, lipopolysaccharides (LPS), lipoteichoic acid (LTA), apoptotic cells, and certain acute phase reactants (15–19). Furthermore, some SRCRD-containing scavenger receptors have been validated as versatile proteins mediating diverse immune responses (20–22).

Most CTLD-containing proteins participate in immunity on the basis of non-self-recognition and binding abilities, which are accomplished by the characteristic CTLDs (7, 23). The structure of CTLDs is maintained by relying on four conserved cysteine residues, which form two disulfide bridges (24). Certain CTLD-containing scavenger receptors play a role in immune defense. LOX-1 and dectin-1 are two scavenger receptors of class E. They are type II transmembrane proteins with CTLDs displaying a scavenger receptor activity (25, 26). Dectin-1 receptor recognizes various bacterial, fungal, and plant carbohydrates. LOX-1 has been implicated in recognizing other ligands, including apoptotic cells and bacteria. The mouse SRCL (belonging to class SR-A4) is a bacteria-binding receptor containing a CTLD that plays a role in host defense (27).

Currently, an increasing number of scavenger receptors and scavenger receptor-like proteins with diverse structures have been characterized in invertebrates. Some of them were demonstrated to participate in immune defense. *Drosophila* Croquemort and *Dm*SR-CI are the class B and C members of the scavenger receptor family, respectively. Both of them are involved in the phagocytosis of bacteria (15, 28). Another insect scavenger receptor *Tm*SR-C plays a pivotal role in phagocytosing fungi and bacteria (29). *Cf* SR, a mollusk SRCRD-containing scavenger receptor, can bind to several kinds of microbial polysaccharides serving as a versatile PRR involved in immune recognition (16). In crustaceans, several scavenger receptors were identified as PRRs participating in antibacterial responses by enhancing phagocytosis of bacteria (30–32). Different from these identified scavenger receptors involved in immunity, a putative transmembrane scavenger receptor-like protein named Bark beetle (Bark) was demonstrated to participate in epithelial cell adhesion and mounting of a core complex of septate junctions (33). To date, few reports demonstrate that Bark and Bark-like proteins play a role in immune defense even though their extracellular regions contain defense-related domains, such as SRCRD and CTLD.

In this study, we identified a Bark-like protein (*Sp*Bark) in an invertebrate animal (mud crab), *Scylla paramamosain*. This protein shares a similar structure with *Drosophila* bark protein possessing three SRCR domains and a CTLD in the extracellular region. To determine whether *Sp*Bark could function as a PRR involved in antibacterial immunity, the binding activities of its four domains to mLDL and microbial polysaccharides were tested, and *Sp*Bark-mediated bacterial clearance activity *in vivo* was also investigated. This study demonstrates the role of *Sp*Bark in antibacterial defense and provides new insights into the diverse biological functions of Bark-like proteins.

## Materials and Methods

### Reagents and Chemicals

Taq DNA polymerases, RNAiso Plus, and First Strand cDNA Synthesis Kit were obtained from TaKaRa (Dalian, China). LPS from *Escherichia coli* 0111:B4 and peptidoglycan (PGN) and LTA from *Staphylococcus aureus* were obtained from Sigma (St. Louis, MO, USA). Trehalose from *Saccharomyces cerevisiae* was purchased from Amresco (Solon, OH, USA).

### Immune Challenges of Mud Crab and Tissue Collection

Mud crabs (~150 g each) purchased from an aquaculture farm in Chongming Country (Shanghai, China) were acclimated in 400 L tanks with aerated seawater for a week (mud crabs are not endangered or protected species, and no special permissions are required). Healthy crabs were randomly selected to investigate the tissue distribution and expression profiles of *SpBark*. For immune stimulation, *Staphylococcus aureus* and *Vibrio parahemolyticus* were cultured overnight in Luria–Bertani medium, collected by centrifugation, and re-suspended in PBS (10 mM Na_2_HPO4, 1.8 mM KH_2_PO4, 140 mM NaCl, and 2.7 mM KCl; pH 7.3). After washing twice with PBS, the resultant suspensions were used as bacterial inocula. Subsequently, 200 μL of each inoculum (2 × 10^7^ CFU in PBS) was injected into the base of the right fifth leg of each crab. The corresponding control was challenged with the same volume of PBS. At each time point after injection (0, 2, 6, 12, 24, 48, and 72 h), hemolymph was drawn from the base of the legs by using a syringe preloaded with ice-cold anticoagulant buffer (0.45 M NaCl, 0.1 M glucose, 30 mM trisodium citrate, 26 mM citric acid, and 10 mM ethylenediaminetetraacetic acid; pH 4.6) (34). The hemocyte pellets were collected for RNA extraction after centrifugation at 800 × *g* for 10 min at 4°C. In addition, other tissues, including the heart, gills, hepatopancreas, stomach, intestine, connective tissue, and muscle, of healthy crabs were dissected; washed with sterile PBS; and collected for RNA extraction. For each tissue sample, at least three crabs were used to eliminate individual differences. Another two batches of previously isolated RNA samples were used to eliminate the differences among batches. All animal experimental procedures were conducted in accordance with the National Institutes of Health's Guide for the Care and Use of Laboratory Animals.

### Total RNA Isolation and First Strand cDNA Synthesis

The total RNA from hemocytes and other collected tissues was isolated with RNAiso Plus, and DNase I (Promega, USA) was added into the extracted total RNA to remove the contaminated DNA. The first stand cDNA was synthesized using the First Strand cDNA Synthesis Kit (TaKaRa, Dalian) in accordance with the manufacturer's instructions.

### *Sp*Bark cDNA Cloning

The original cDNA sequence of *SpBark* was obtained through high-throughput transcriptome sequencing with an RNA mixture isolated from the hemocytes, gill, and hepatopancreas, and it was further confirmed by PCR with five pairs of primers (Table 1). The PCR was performed under the following parameters: 95°C for 5 min; 35 cycles of 94°C for 30 s, 55°C for 30 s, and 72°C for 60 s; and finally 72°C for 10 min. The DNA products were separated by agarose gel electrophoresis, subcloned into a pMD-19T vector, and sequenced via a commercial company (Sangon, China).

**Table 1:** Primers used in this study.

**Primers**	**Sequence (5^′^-3^′^)**
**cDNA cloning**	
*Sp*BarkF1	TGGTGGTGGTTGTGGTGGTG
*Sp*BarkR1	CCTAAACATGTTTTGGCCAA
*Sp*BarkF2	AATAATCACCGTTTGGCCAT
*Sp*BarkR2	GGAAGGCCACCTGGTCCCCT
*Sp*BarkF3	CTACCACCTCTAGTAGTTACG
*Sp*BarkR3	GGAGTTTGATGTTCTCCATG
*Sp*BarkF4	AGTCTACCCTTCCATTTTGG
*Sp*BarkR4	CAGAGGGTCTGAAGCCCAGGC
*Sp*BarkF5	GCAGTGGCAGCCATTGCTAT
*Sp*BarkR5	ATGAAGTAAACGCTTTCTAAT
**Real-time PCR**	
*Sp*BarkRF	TCACACGCCGCAGGATAAT
*Sp*BarkRR	GCTGAGAAGAGTAACCAGGAGGA
**18S rRNA**	
18SF	CAGACAAATCGCTCCACCAAC
18SR	GACTCAACACGGGGAACCTCA
**Protein expression**	
SRCRD1EF	TACTCAGGTACCCTGCGCATAGTGGACGGCC
SRCRD1ER	TACTCACTCGAGTTAGTCACAGTCCACGCCGAGGT
SRCRD2EF	TACTCAGGTACCGTTAAGCTAGTGGGTGGCAG
SRCRD2ER	TACTCACTCGAGTTAGTAGCAACGGATGCCCACAT
SRCRD3EF	TACTCAGGTACCGTGAGACTGTGTGTGGAAG
SRCRD3ER	TACTCACTCGAGTTATCCACAAGAGATGTACACAAAG
CTLDEF	TACTCAGGTACCTGTGAACCAGGCTACACCT
CTLDER	TACTCACTCGAGTTACTCGCAGATAAATGGCAGCT
**RNAi**	
*Sp*BarkiF	GCGTAATACGACTTATGGGACCACCTCACACAACAGTCT
*Sp*BarkiR	GCGTAATACGACTTATGGGTTCATCTGCATCAGACTCGC
GFPiF	GCGTAATACGACTTATGGGTGGTCCCAATTCTCGTGGAAC
GFPiR	GCGTAATACGACTTATGGGCTTGAAGTTGACCTTGATGCC

*Underlined nucleotides indicate the locations of restricted endonucleases*.

### Bioinformatics Analyses

The identities of *Sp*Bark with other Bark-like proteins were revealed using the online Basic Local Alignment Search Tool Program (BLASTP) (http://blast.ncbi.nlm.nih.gov/Blast.cgi). The domain architecture was predicted using simple modular architecture research tool (SMART) (http://smart.embl-heidelberg.de). Multiple alignment was generated through the ClustalX 2.0 program (http://www.ebi.ac.uk/tools/clustalw2) and GENEDOC software. The putative amino acid sequence was deduced and predicted on http://web.expasy.org/translate/. The signal peptide was predicted with SignalP (http://www.cbs.dtu.dk/services/SignalP/). The pI and molecular weight (MW) were calculated on http://web.expasy.org/compute_pi/. MEGA 7.0 was used to construct a neighbor-joining tree, and the bootstrap of 1,000 was selected to assess reliability.

### Quantitative Real-Time PCR (qRT-PCR)

qRT-PCR was conducted to investigate the relative expression level of *Sp*Bark in a real-time thermal cycler Quantstudio 6 Flex (ABI, USA) according to a previous protocol (35). A pair of primers (*Sp*BarkRF and *Sp*BarkRR) was designed to generate an amplicon of *Sp*Bark. Another pair of primers (18SRF and 18SRR) was synthesized to produce a 121-bp fragment of 18S rRNA as reference. qRT-PCR was programed as follows: an initial denaturation at 94°C for 3 min; 40 cycles of 94°C for 10 s and 60°C for 1 min; and finally a melting curve analysis from 65 to 95°C. The total volume of reaction mixture was 20 μL (10 μL of 2 × SYBR Premix Ex Taq, 2 μL of cDNA, and 4 μL of each primer). All tests were performed thrice with individual templates. The algorithm of 2^−Δ*CT*^ was used to investigate tissue distribution of *Sp*Bark. To analyze the time-course expression profiles, the formula of 2^−Δ*ΔCT*^ was used to normalize the data in two steps. First, the expression of *Sp*Bark was normalized to the reference gene (18S rRNA) in the same sample. Second, the relative expression of *Sp*Bark in the experimental sample was normalized to that in the control sample. Unpaired Student's *t*-test was used to analyze significant differences (^*^P < 0.05; ^**^P < 0.01).

### Recombinant Expression and Purification

To explore the potential immune function of *Sp*Bark, four predicted functional domains (SRCRD1, SRCRD2, SRCRD3, and CTLD) were overexpressed using an *E*. *coli* expression system. On the basis of the *Sp*Bark cDNA sequence, four primer pairs (SRCRD1EF and SRCRD1RF, SRCRD2EF and SRCRD2ER, SRCRD3EF and SRCRD3ER, and CTLDEF and CTLDER) were designed to produce DNA fragments encoding SRCRD1, SRCRD2, SRCRD3, and CTLD, respectively (Table 1). These DNA fragments were digested and then inserted into the pET32a expression vectors. The constructed plasmids were transformed into competent *E*. *coli* cells for overexpression. Recombinant proteins were induced by adding isopropyl-β-d-thiogalactoside (IPTG) to a final concentration of 0.5 mM at 37°C for 6 h and purified using High Affinity Ni-NTA Resin (GenScript, Nanjing) in accordance with the manufacturer's instructions.

### Microorganism-Binding Assay

Nine kinds of microorganisms, including four Gram-negative bacteria (*Vibrio harveyi, V. parahemolyticus, V. alginolyticus*, and *E*. *coli*), three Gram-positive bacteria (*S*. *aureus, Bacillus subtilis*, and *B. megaterium*), and two fungi (*Candida albicans* and *Pichia pastoris*), were used to investigate the microorganism-binding activities of SRCRD1, SRCRD2, SRCRD3, and CTLD. The experiment was performed following a protocol used in our previous study (36). In brief, the microorganisms (1 × 10^8^ CFU) were incubated in 1 mL of each recombinant protein (200 μg/mL) with gentle rotation for 30 min at room temperature. The microorganisms were pelleted, washed four times with 1 mL of TBS (150 mM NaCl, 10 mM Tris–HCl, pH 7.5), and eluted with 200 μL of 7% SDS solution with mild agitation for 5 min at room temperature. The microorganism pellets were rewashed thrice with 1 mL TBS. Finally, the elution and pellet of each microorganism were sampled and analyzed on 12.5% SDS-PAGE. The recombinant proteins (SRCRD1, SRCRD2, SRCRD3, and CTLD) were sampled as the positive controls. The binding activity to microorganisms was determined by Western blot. After the proteins were transferred onto a PVDF membrane, the membrane was blocked with 4% bovine serum albumin (BSA) in TBS and incubated with peroxidase-conjugated monoclonal antibody against His-tag (GenScript, Nanjing). The target proteins were visualized with an ECL Western blot reagent kit.

### Binding Activity of Recombinant Proteins to Microbial Polysaccharides

Enzyme-linked immunosorbent assay (ELISA) was performed to test the binding abilities of four domain proteins (SRCRD1, SRCRD2, SRCRD3, and CTLD) to microbial polysaccharides and acetylated LDL (ac-LDL) following a previously described protocol with slight modifications (37). Microbial polysaccharides, including PGN and LTA from *S. aureus*, LPS from *E. coli* 0111:B4, and β-glucan from *Laminaria digitata*, together with human ac-LDL were used in this study. Each well of the microtiter plate was incubated with 100 μL of ac-LDL (50 μg/mL) at 4°C overnight or microbial polysaccharide (20 μg/mL) at 37°C until the plate came to desiccation. Wells incubated with 100 μL of distilled water were employed as blank control. Each well was blocked with BSA (1 mg/mL, 200 μL) at 37°C for 2 h and washed four times with TBST (0.05% Tween 20 in TBS). Subsequently, a series of diluted recombinant protein SRCRD1, SRCRD2, SRCRD3, CTLD, or TRX (0–5 μM in TBS containing 0.1 mg/mL BSA) was added to polysaccharide-coated plates. TRX was used as negative control. The serially diluted recombinant proteins supplemented with 5 mM of CaCl_2_ were added to ac-LDL-coated plates. After incubation at room temperature for 3 h, the plates were rinsed with TBST four times. Each well was then incubated with 100 μL of peroxidase-conjugated mouse monoclonal anti-His antibody (1:5,000 dilution in TBS with 0.1 mg/mL BSA) at 37°C for 2 h. The color reaction was developed with 0.01% 3,3′,5,5′-tetramethylbenzidine (Sigma, USA) and stopped by adding 2 M H_2_SO_4_. The absorbance was read at 405 nm on a Spark 10 M microplate reader (Tecan, Switzerland). The assay was conducted in triplicate. For a competitive binding assay, purified recombinant CTLD was diluted to a final concentration of 0.25 μM and pre-incubated with different LPS concentrations (0.0005, 0.0025, 0.005, 0.025, 0.05, 0.25, 0.5, and 2.5 μM) by gentle shaking at room temperature for 2 h (38). Subsequently, the pre-incubated CTLD protein (100 μL per well) was added to the plates coated with LPS. The following steps were the same as those in ELISA above. The competitive binding assay was repeated three times.

### Agglutination Assay

The microorganisms used in microorganism-binding assay were applied for agglutination assay following a method described by (36). Microorganisms were harvested at the mid-logarithmic growth phase by centrifugation at 5,000 × g for 5 min, re-suspended in TBS after washing three times, and finally adjusted to 2 × 10^8^ cells/mL (for bacteria) or 2 × 10^7^ cells/mL (for fungi). The microorganism suspensions were mixed with equal volume (100 μL) of diluted recombinant protein (SRCRD1, SRCRD2, SRCRD3, CTLD, or TRX) in TBS at the protein concentration range of 0.48–15.2 μM with or without 10 mM CaCl_2_ at 28°C for 30 min. TRX tag protein served as the negative control. Agglutination was observed under a light microscope.

### RNA Interference

A partial *Sp*Bark DNA fragment, which covered the *Sp*Bark CTLD, was amplified by PCR with specific primers linked to the T7 promoter (Table 1) and used as the template to generate ds*Sp*Bark (*Sp*Bark dsRNA) with T7 RiboMAX™ Express RNAi System (Promega, USA). The ds*GFP* (EGFP dsRNA) was synthesized as control with primers listed in Table 1. To obtain better RNA interference efficiency, the crabs with an average weight of ~25 g were selected and divided into two groups (four crabs in each group). Each crab in the experimental group was injected with 25 μg of ds*Sp*Bark into the base of the fourth leg. The crabs in the control group were injected with an equal amount of ds*GFP*. A second dsRNA injection was performed 24 h later in the same way. Hemocytes were collected from each group at 40 and 48 h after the first dsRNA injection. RNAi efficiency was assessed by qRT-PCR using the total RNAs from hemocytes with a pair of primers located outside the above DNA fragment (Table 1). Unpaired *t*-test was used to analyze significant differences (^*^P < 0.05; ^**^P < 0.01).

### Bacterial Clearance Assay

*V. parahemolyticus* was incubated with recombinant CTLD protein and then injected into crabs to determine whether coating bacteria with CTLD could influence bacterial clearance. Approximately 500 μL of CTLD protein in PBS (400 μg/mL) was mixed with an equal volume of *V*. *parahemolyticus* (1 × 10^8^ CFU) with gentle rotation at 28°C for 60 min. The same number of *V*. *parahemolyticus* incubated with TRX tag protein (400 μg/mL) and equal volume of PBS were also prepared to serve as controls. Mud crabs were divided into three groups (four crabs in each group). After incubation, the mixtures (200 μL) were injected into crabs. At each time point post-injection (5, 15, and 30 min), hemolymph (200 μL) was collected from crabs and mixed with an equal volume of anticoagulant buffer. After serial dilution with PBS, the samples (100 μL) were plated onto the LB plates. These plates were then incubated at 37°C overnight, and the number of bacterial clones on the plates was counted, which was used to determine the number of residual bacteria in hemolymph. After validating that *Sp*Bark expression could be silenced by injection of ds*Sp*Bark, we investigated whether *Sp*Bark knockdown could affect bacterial clearance. Each crab was injected with 200 μL of *V*. *parahemolyticus* suspension (2 × 10^7^ cells) at 40 h after injection with ds*Sp*Bark or ds*GFP*. After mock PBS injection, the crabs were treated with an equal amount of bacteria in the same way. The number of residual bacteria in hemolymph was calculated using the method described above. The experiments were performed three times, and the results presented the mean of three individual experiments. Unpaired *t*-test was used to analyze significant differences (^*^P < 0.05; ^**^P < 0.01).

### Fluorescent Labeling of Bacteria and Phagocytosis Assay

The labeling of *V*. *parahemolyticus* with fluorescein isothiocyanate isomer I (FITC, Sigma) was conducted as described earlier (32). Overnight cultures of *V*. *parahemolyticus* were washed twice with PBS, re-suspended in carbonate buffered saline (pH 9.5) containing 0.2 mg/mL FITC, and then incubated at 25°C for 1 h. Subsequently, the FITC-labeled bacteria were rewashed four times to remove dissociated FITC and then re-suspended in PBS (~1 × 10^9^ CFU/mL). Approximately 200 μL of FITC-labeled bacteria was injected into the mud crabs at 40 h after injections with ds*Sp*Bark and ds*GFP*. Hemolymph was collected at 30 min after bacterial injection using a syringe preloaded with anticoagulant containing 4% paraformaldehyde. After incubation at 4°C for 15 min, the hemocytes were centrifuged and re-suspended in anticoagulant. Trypan blue (2 mg/mL, Sigma) was added into the hemocyte suspension and incubated for 5 min to quench the fluorescence of non-phagocytized bacteria. After washing with PBS twice, the fixed hemocytes were dropped onto poly-L-lysine-coated glass slides, and the phagocytized bacteria were evidently detected under a fluorescence microscope (Nikon Eclipse Ti-E, Japan). A total of 600 cells (each group) were counted to determine the phagocytic percentage, which was defined here as [hemocytes ingesting bacteria/all cells observed or tested] × 100%. The phagocytosis assay was conducted three times. The data were subjected to Student's *t*-test, and differences with *P* < 0.05 were considered statistically significant.

## Results

### cDNA Cloning of *Sp*Bark

**Figure 1:**
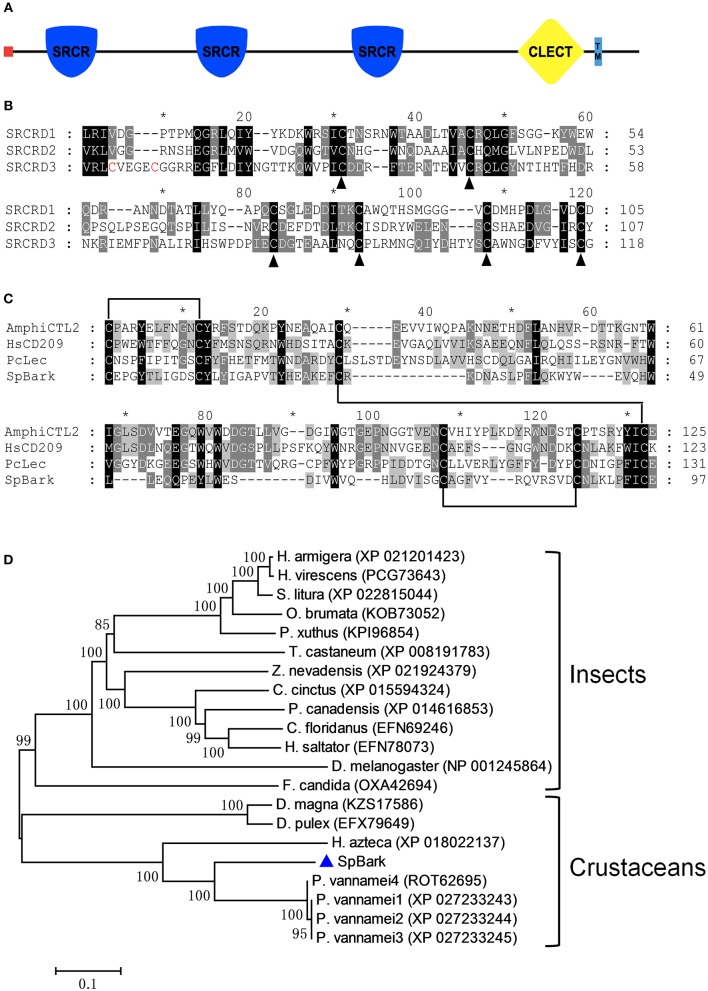
Architecture and alignments of *Sp*Bark domains and phylogenetic analysis. **(A)** Schematic of *Sp*Bark domains predicted with online software SMART. A signal peptide, three SRCRDs (from *N*-terminus to *C*-terminus: SRCRD1, SRCRD2, and SRCRD3), a CTLD, and a transmembrane region were predicted and marked with different shapes and colors. **(B)** Alignment of three SRCRDs. These three domains show low similarity, but six cysteine residues in these sequences were conserved except the additional two cysteine residues at the *N*-terminus of SRCRD3. The alignment was marked with an asterisk every 10 amino acids. **(C)** Alignment of CTLDs. The alignment was conducted with CTLDs from *Sp*Bark, *Pc*lec2, human CD209 (AAI10616), and amphioxus C-type lectin (ABY54815). **(D)** Phylogenetic analysis of retrieved Bark or Bark-like proteins from mud crab and other species on the basis of BLASTP results. MEGA 7.0 software was used to generate the neighbor-joining tree with a bootstrap of 1,000. The bar shows the relative distance of genetic variation. *Sp*Bark was marked with a blue triangle.

The full-length cDNA sequence of *Sp*Bark is 10,053 bp long, including a 24-bp 5′ untranslated region (UTR), a 1,437-bp 3′ UTR containing a poly(A) tail, and an 8,592-bp open reading frame encoding a polypeptide of 2,863 amino acids (GenBank Accession No. MH595537) (Figure 1S). The deduced protein had an 18-amino-acid signal peptide at the *N*-terminus and a transmembrane region close to the C-terminus. Its mature peptide had an estimated MW of 320.8 kDa and a theoretical pI of 5.58. Three putative SRCR domains, including SRCRD1 (Leu^106^ to Asp^210^), SRCRD2 (Val^976^ to Tyr^1082^), and SRCRD3 (Val^1797^ to Gly^1914^), and a following CTLD (Cys^2512^ to Glu^2608^) were found in this deduced protein (Figure 1A). The pIs of SRCRD1, SRCRD2, SRCRD3, and CTLD were 5.12, 4.58, 5.48, and 4.95, respectively.

### Similarity and Phylogenetic Analyses

The BLASTP search analysis demonstrated that *Sp*Bark shared 71–73% identity with four Bark beetle-like proteins from *Penaeus vannamei* (four isoforms: ROT62695, XP_027233243, XP_027233244, and XP_027233245). *Sp*Bark also shared 59% identity with *Hyalella azteca* Bark-like protein (XP_018022137) but no more than 42% identity with other putative Bark-like proteins. The alignment of three SRCR domains revealed that six cysteine residues in these SRCR domains, which might be responsible for forming three disulfide bonds to stabilize the dimensional structure of SRCR domains, were well-conserved even though these three amino acid sequences shared a low identity of 29.4% (Figure 1B). Another two cysteine residues at the N-terminus of SRCRD3 were also found, suggesting that SRCRD3 may have a different structure from SRCRD1 and SRCRD2. Alignment of the CTLDs of lectins from mud crab, shrimp, human, and amphioxus showed that four cysteine residues (Cys49, Cys127, Cys142, and Cys150), which can form two disulfide bonds to stabilize the structure of classic CTLDs, were well-conserved in *Sp*Bark CTLD. Two additional cysteine residues, which may form another disulfide bond, were found at the N-terminus of each domain. Moreover, some remarkable amino acid motifs, such as EPN (Glu-Pro-Asn), QPD (Gln-Pro-Asp), and WND (Trp-Asn-Asp), responsible for carbohydrate binding were not found in *Sp*Bark CTLD. These results suggested that *Sp*Bark CTLD was unique with different sequence information from the classic CTLDs (Figure 1C).

On the basis of BLASTP results, a phylogenetic tree was constructed using Bark-like proteins to analyze the evolutionary relationships among them. In this tree, Bark-like proteins were divided into two large clusters (Figure 1D). *Sp*Bark together with other crustacean Bark-like proteins was grouped into the crustacean cluster, and the other Bark-like proteins were clustered into the insect group. *Sp*Bark and four shrimp Bark-like proteins formed a meaningful subcluster with the node value of 99, indicating that Bark-like proteins may widely exist in decapod crustaceans possessing special biological function different from the ones in insects.

### *Sp*Bark Was Widely Distributed in Mud Crabs and Upregulated by Bacterial Challenges

**Figure 2:**
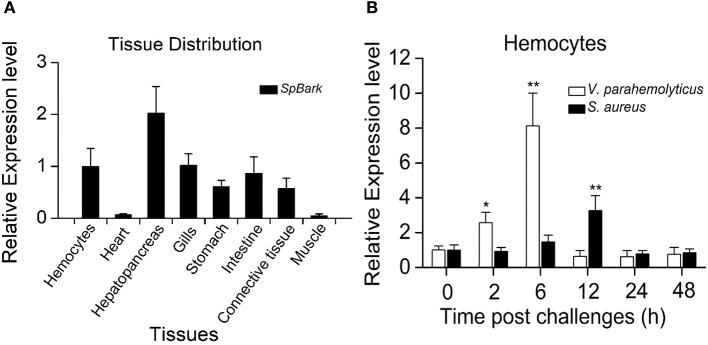
Tissue distribution and expression profiles of *Sp*Bark. **(A)** Tissue distribution of *Sp*Bark was analyzed by qRT-PCR using 18S rRNA as the internal control. **(B)** Expression profiles of *Sp*Bark in hemocytes at different time points after challenges with *Staphylococcus aureus* and *Vibrio anguillarum*. Significant differences are indicated with asterisks (^*^P < 0.05; ^**^P < 0.01).

qRT-PCR analysis revealed that *Sp*Bark was highly expressed in the hepatopancreas, hemocytes, gills, intestine, and stomach, and it was detected in the heart and muscle at a low expression level (Figure 2A). The temporal expression profiles of *Sp*Bark in hemocytes after bacterial challenges were further investigated to explore whether this gene participated in immune defense. *Sp*Bark was remarkably increased 2–6 h after challenge with *V. parahemolyticus* and reached the highest level (~7 fold increase) at 6 h post-injection. With regard to the challenge with *S. aureus*, significant induction (~2 fold increase) was observed at 12 h post-injection (Figure 2B). These results suggested that *Sp*Bark was involved in antibacterial responses of mud crabs.

### Four Structural Domain Proteins Were Recombinantly Expressed and Purified

**Figure 3:**
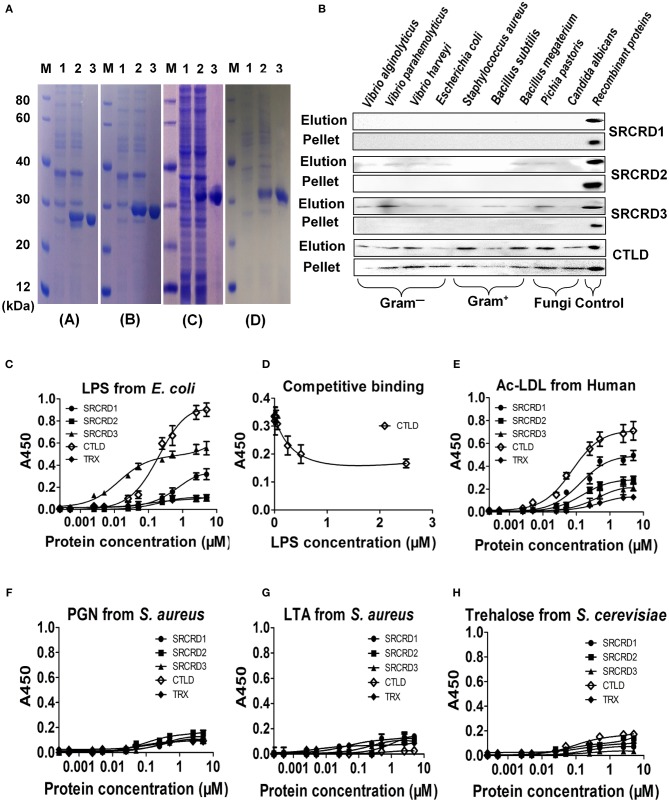
Purification and binding activities of four functional domains of *Sp*Bark. **(A)** All four functional domains (SRCRD1, SRCRD2, SRCRD3, and CTLD) were expressed in *E. coli* and then purified. Lane M, protein marker; lane 1, total proteins of *E. coli* without IPTG induction; lane 2, total proteins of *E. coli* with IPTG induction; lane 3, the purified recombinant proteins. **(B)** Binding activity of recombinant SRCRD1, SRCRD2, SRCRD3, or CTLD to different microorganisms. The binding activities of these four proteins were confirmed by Western blot, and recombinant proteins SRCRD1, SRCRD2, SRCRD3, and CTLD were sampled as the corresponding positive controls. Elution, the supernatant was harvested after centrifugation of microorganisms treated with 7% SDS solution; pellet, the eluted microorganism was obtained after three washes with PBS. **(C–H)** Microbial polysaccharide-binding activities were investigated using ELISA. LPS from *E. coli* (C and D), acetylated LDL from humans (E), PGN from *S. aureus* (F), LTA from *S. aureus* (G), and trehalose from *S. cerevisiae* (H) were used to coat plates. Recombinant proteins SRCRD1, SRCRD2, SRCRD3, CTLD, and TRX (negative control) were serially diluted and added into the coated plates. **(D)** Recombinant CTLD pre-incubated with different amounts of LPS was added for the competitive binding activity assay. Results were obtained on the basis of three independent repeats.

The recombinant proteins of the four structural domains of *Sp*Bark were overexpressed in soluble form and then purified by Ni-NTA His Bind Resin. The predicted MWs of recombinant SRCRD1, SRCRD2, SRCRD3, and CTLD (each fused with a ~16 kDa Trx tag) were approximately 27.8, 28.0, 29.7, and 27.4 kDa, respectively. The position of each purified protein is roughly in agreement with the size of the corresponding recombinant protein (Figure 3A).

### *Sp*Bark CTLD Exhibited the Strongest Binding Activity to Microorganisms

The microbial cell-binding activities of the four structural domain proteins were tested by Western blot. The recombinant protein was detected in pellets representing that it possessed strong binding activity, and the one in elution meant that it exhibited weak binding toward microorganisms. On the basis of these criteria, CTLD exhibited strong binding activity to all tested microorganisms, including four Gram-negative bacteria (*V. harveyi, V*. *parahemolyticus, V. alginolyticus*, and *E. coli*), three Gram-positive bacteria (*S. aureus, B. subtilis*, and *B. megaterium*), and two fungi (*C. albicans* and *P. pastoris*). By contrast, SRCRD2 and SRCRD3 only exhibited weak binding activities to different kinds of microorganisms, and SRCRD1 possessed no evident binding activity to microorganisms (Figure 3B). These results implied that *Sp*Bark CTLD was the major functional domain involved in bacterial recognition.

### *Sp*Bark CTLD Displayed the Most Potent Binding Activity to LPS and ac-LDL

The binding activities of *Sp*Bark SRCRD1, SRCRD2, SRCRD3, and CTLD to ac-LDL and several kinds of polysaccharides were investigated by performing ELISA. In the ac-LDL binding assay, only three of the four domain proteins (SRCRD1, SRCRD2, and CTLD) exhibited ac-LDL-binding activities in varying degrees (Figure 3E). Among them, *Sp*Bark CTLD possessed the strongest binding activity, and SRCRD1 displayed more potent binding activity than SRCRD2. Given that the binding activity to mLDL is a marked property of scavenge receptors, *Sp*Bark possessing ac-LDL binding activity suggested that it might function as a scavenger receptor in mud crab. In addition, SRCRD1, SRCRD3, and CTLD exhibited notable LPS-binding activities, but none of the four domain proteins displayed apparent binding activities to the other tested polysaccharides, such as LTA, PGN, and trehalose; this finding indicated that the microorganism-binding activity exhibited by CTLD might be through binding to LPS or other untested components on the cell surface (Figures 3C,F–H). This study also revealed that CTLD displayed a much stronger LPS-binding activity than SRCRD1 and SRCRD3. The specific LPS-binding activity exhibited by CTLD was further determined by a competitive binding assay. The results demonstrated that the binding ability of CTLD to immobilized LPS on the microplates was significantly reduced by pre-incubation of CTLD with LPS, and the binding activity to immobilized LPS became weak with increasing amount of LPS to pre-incubate CTLD (Figure 3D). These results suggested that the *Sp*Bark binding activity to Gram-negative bacteria was mainly through binding LPS with its CTLD.

### *Sp*Bark CTLD Agglutinated Bacteria in the Presence of Ca^2+^

**Figure 4:**
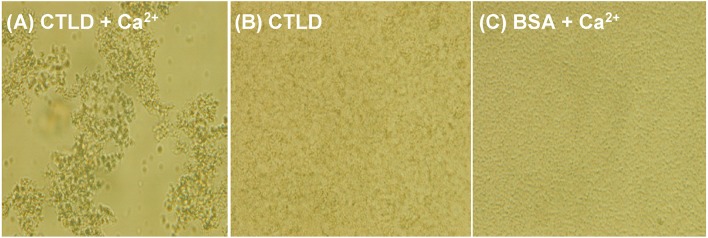
Agglutination of *V. parahemolyticus* induced by *Sp*Bark CTLD in the presence of Ca^2+^. *V. parahemolyticus* was incubated with *Sp*Bark CTLD with **(A)** or without Ca^2+^ **(B)**. BSA plus Ca^2+^ was used as the negative control **(C)**. Agglutination was observed through light microscopy.

The agglutination activities of SRCRD1, SRCRD2, SRCRD3, and CTLD were investigated to explore whether they were involved in bacterial agglutination. Results demonstrated that only *Sp*Bark CTLD of these four proteins exhibited apparent agglutination activity in the presence of Ca^2+^ (Figure 4). The agglutination activities of CTLD to different microorganisms greatly varied. Table 2 shows that the minimal agglutinating concentration (MAC) of CTLD to Gram-positive bacteria *S. aureus* and *B. megaterium* was the same as that to fungus *P. pastoris*, which was lower than the MACs to Gram-negative bacteria. This finding indicates that CTLD possessed stronger agglutinating abilities to Gram-negative bacteria than the other tested microorganisms. *Sp*Bark CTLD could agglutinate bacteria, suggesting that it can function like many common CTLDs acting as a binding site of PRR.

**Table 2:** Agglutination activities of four domains of *Sp*Bark in the presence of Ca^2+^.

**Microorganisms**	**MAC (μM)**			
	**SRCRD1**	**SRCRD2**	**SRCRD3**	**CTLD**
**Gram^+^**				
*Staphylococcus aureus*	–	–	–	3.8–7.6
*Bacillus subtilis*	–	–	–	–
*Bacillus megaterium*	–	–	–	3.8–7.6
**Gram^−^**				
*Vibrio alginolyticus*	–	–	–	1.9–3.8
*Vibrio parahemolyticus*	–	–	–	0.95–1.9
*Vibrio harveyi*	–	–	–	0.95–1.9
*Escherichia coli*	–	–	–	0.95–1.9
**Fungi**				
*Pichia pastoris*	–	–	–	3.8–7.6
*Candida albicans*	–	–	–	–

*MAC was defined as the lowest protein concentration showing apparent agglutination compared with the negative control. “–” means no evident agglutination was observed when the protein concentration was 7.6 μM*.

### *Sp*Bark CTLD Promoted *V. parahemolyticus* Clearance in Hemolymph

**Figure 5:**
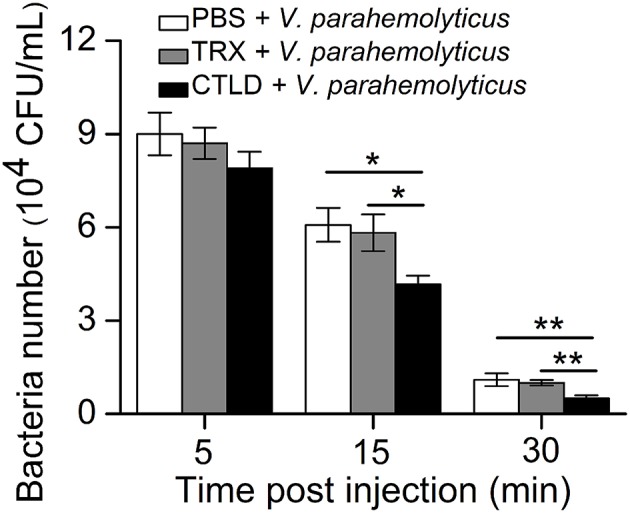
Promotion of bacterial clearance by injection with *Sp*Bark CTLD protein. The ability to clear bacteria (*V. parahemolyticus*) in hemolymph was increased by the “overexpression” of CTLD protein with TRX tag protein and PBS as controls. The results presented the mean of three individual experiments. Asterisks indicate the significant differences (^*^P < 0.05; ^**^P < 0.01).

Given that *Sp*Bark CTLD exhibited specific binding abilities to LPS and Gram-negative bacteria, we investigated whether this protein facilitated bacterial clearance in crabs through its binding activity. *V. parahemolyticus* pre-incubated with CTLD, Trx protein, or PBS was injected into healthy crabs. The number of bacteria *in vivo* was significantly decreased at 15 min post-injection by pre-incubating *V. parahemolyticus* with CTLD compared with that treated with Trx or PBS; however, no significant differences in bacterial numbers were found at 5 min post-injection among these three groups. At 30 min after the injection of bacteria treated with CTLD, an extremely significant decrease in the number of bacteria (*P* < 0.01) in crabs was observed compared with that in control crabs (Figure 5). These results revealed that pre-incubation of *V. parahemolyticus* with CTLD would facilitate bacterial clearance *in vivo*.

### Knockdown of *Sp*Bark Reduced Bacterial Clearance in Mud Crab

**Figure 6:**
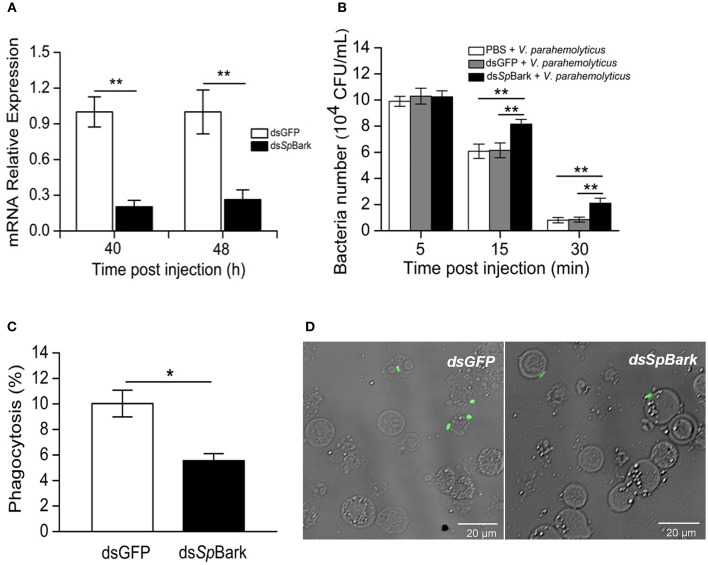
RNAi of *Sp*Bark suppresses bacterial clearance in hemolymph by modulating hemocyte phagocytosis. **(A)** Knockdown of *Sp*Bark. The mRNA expression level of *Sp*Bark in hemocytes after injection with ds*Sp*Bark or ds*GFP* was analyzed by qRT-PCR. **(B)** Suppression of bacterial clearance by *Sp*Bark dsRNA injection. Bacterial clearance ability in hemolymph after *Sp*Bark knockdown was evaluated. Either ds*GFP* or PBS was injected and served as control. **(C)** Phagocytosis of FITC-labeled *V. parahemolyticus* by hemocytes in crabs. At 40 h after the first treatment with ds*Sp*Bark, FITC-labeled *V. parahemolyticus* was injected into crabs. Hemocytes were collected 30 min later, and then hemocytes and *V. parahemolyticus* cells (green) were observed and counted under a fluorescence microscope. The ds*GFP*-treated crabs were used as control. **(D)** The phagocytosis rate was calculated using images captured by the fluorescence microscope, and a total of 600 cells in each group were counted. The results were shown as the means of three individual experiments. Asterisks indicate the significant differences (^*^P < 0.05; ^**^P < 0.01).

To investigate the *in vivo* function of *Sp*Bark, RNAi of *Sp*Bark and bacterial clearance assays were performed. qRT-PCR analysis indicated that the transcripts of *Sp*Bark in hemocytes significantly declined at 40 and 48 h after the first injection of ds*Sp*Bark (Figure 6A). This result indicated that the injection of *Sp*Bark dsRNA into crabs could remarkably suppress *Sp*Bark expression. After *Sp*Bark knockdown, *V. parahemolyticus* was injected into crabs, and the residual number in hemolymph was counted to assess the bacterial clearance ability. As shown in Figure 6B, the residual number of *V. parahemolyticus in vivo* significantly increased at 15 and 30 min post-injection compared with that in ds*GFP* or PBS group. This result suggested that the bacterial clearance ability in hemolymph of *Sp*Bark-silenced crabs was severely impaired.

### *Sp*Bark Promoted Phagocytosis of Bacteria

To reveal the mechanism by which *Sp*Bark facilitates bacterial clearance, bacterial phagocytosis assay was conducted. After *Sp*Bark was silenced, FITC-labeled bacteria were injected into crabs. Hemocytes were collected to determine the phagocytosis rate. The results showed that the phagocytic rate of hemocytes in *Sp*Bark-silenced crabs was significantly lower than that in control crabs: the phagocytosis rate was decreased by ~45% by knockdown of *Sp*Bark (from 9.7 to 5.3%) (Figures 6C,D). This result suggests that *Sp*Bark modulated hemocyte phagocytosis of *V. parahemolyticus*.

## Discussion

Scavenger receptors are widely distributed in invertebrate and vertebrate animals exhibiting diverse biological functions through binding to various ligands (8). In the present study, we characterized a novel scavenger receptor-like protein in *S. paramamosain*, which shared high identity with Bark or Bark-like protein and thus designated as *Sp*Bark. *Sp*Bark CTLD displayed the strongest binding activity to ac-LDL and LPS. Knockdown of *Sp*Bark remarkably suppressed the clearance of bacteria *in vivo* and phagocytosis of bacteria. These findings suggested that *Sp*Bark might function as a potential PRR playing an important role in immune defense against Gram-negative bacteria.

Recently, a consensus definitive classification of scavenger receptors was proposed, and the scavenger receptors of mammals were categorized into 12 classes on the basis of their sequence structure and biological functions (11). Though *Sp*Bark protein contains SRCRD and CTLD, which are characteristic domains present in class A, E, and I scavenger receptors, clustering this protein into any denoted class of mammalian scavenger receptors seems difficult. No consensus nomenclature system for invertebrate scavenger receptors and definitive criteria to define scavenger receptors exist. The scavenger receptor-like protein “Bark” was first characterized in *Drosophila*, which has the signature structural domains (SRCRD and CTLD) present in the mammalian scavenger receptors. However, it was first identified as a scaffold protein involved in mounting of the core complex of septate junctions (33). Thus, most Bark homologs from other species were displayed as Bark or Bark-like proteins in BLASTP search results because no new biological function was further reported regarding *Drosophila* Bark and its homologs. Considering that *Sp*Bark shared a similar distribution pattern of structural domains (SP at N-terminus, three SRCRDs, one CTLD, and TM region at C-terminus) with *Drosophila* Bark and had 40% identity at the protein level, we named this scavenger receptor-like protein as a new Bark-like protein in mud crab. Bark homologs were also found in other invertebrate animals. A phylogenetic tree constructed using invertebrate Bark homologs revealed that *Sp*Bark together with the other crustacean Bark proteins formed a unique meaningful cluster. This finding suggested that crustacean Bark proteins had a close evolutionary relationship and might possess similar biological functions.

Most C-type lectins possess binding activities to microbial polysaccharides and exhibit agglutination activity with their CTLDs (7, 23). Different from the classic CTLDs with four cysteine residues forming two disulfide bonds, *Sp*Bark CTLD was a “long form” CTLD containing six cysteine residues, which could generate three disulfide bonds. This finding suggested that it might form a distinct structure displaying special functions. Our study revealed that *Sp*Bark CTLD was different from certain classic CTLDs displaying broad polysaccharide-binding abilities (7, 39, 40), which only exhibited binding activity to LPS but not to other tested polysaccharides. Apart from the possible effect of the distinct structure on its biological function, we speculated that this limited polysaccharide-binding activity partially originated from the lack of some signature motifs (e.g., EPN, QPD, and WND) essential for polysaccharide-binding activity in *Sp*Bark CTLD. The specific binding activity to LPS may rely on certain mutant carbohydrate-binding motifs in *Sp*Bark CTLD because some mutant or unknown carbohydrate-binding motifs present in reported CTLDs had been shown to be involved in polysaccharide-binding activity (7). *Sp*Bark CTLD exhibited notable agglutination activity against bacteria and a fungus in the presence of Ca^2+^, which was the basic function of classic CTLDs. This finding suggested that it could function similar to the classic CTLDs acting as the binding regions of recognition receptors. Given that the *Sp*Bark protein was a macromolecule with three SRCRDs and one CTLD, we speculated that the major biological function of *Sp*Bark might be displayed through these functional domains. *Sp*Bark CTLD exhibited a much stronger binding activity to LPS than the other SRCRDs, indicating that this CTLD was the key recognition domain of *Sp*Bark.

Although *Drosophila* Bark plays a role in cell adhesion by mounting of the core complex to facilitate the maturation of septate junctions (33), whether this protein and its homologs in other species are involved in immune defenses remains unclear. In this study, *Sp*Bark was upregulated in hemocytes by bacterial challenges, suggesting that it might be an important component implicated in immune defense against bacteria. In addition, though many mammalian scavenger receptors are distributed in specific cells or tissues, *Sp*Bark was highly expressed in the hemocytes, hepatopancreas, gills, and intestine, which are the major immune tissues or organs in crustaceans. Among them, hemocytes can remove the invading pathogens through different mechanisms, such as phagocytosis, nodulation, and encapsulation, playing key roles in immune defense of circulating hemolymph. The hepatopancreas, equivalent to the liver of mammals or fat body of insects, was considered the key immune tissue. Gills and intestine easily contacted potential pathogens existing in water environment and intestinal contents, which might be involved in the initial defenses against external pathogens. Considering that the functional domains of reported mammalian scavenger receptors and *Drosophila* Bark were located at the extracellular regions (33), the SRCRDs and CTLD of *Sp*Bark might be the extracellular domains because *Sp*Bark and *Drosophila* Bark shared the same distribution pattern of functional domains. *Sp*Bark ubiquitously distributed in immune tissues may facilitate the recognition and elimination of bacteria via its extracellular CTLD.

**Figure 7:**
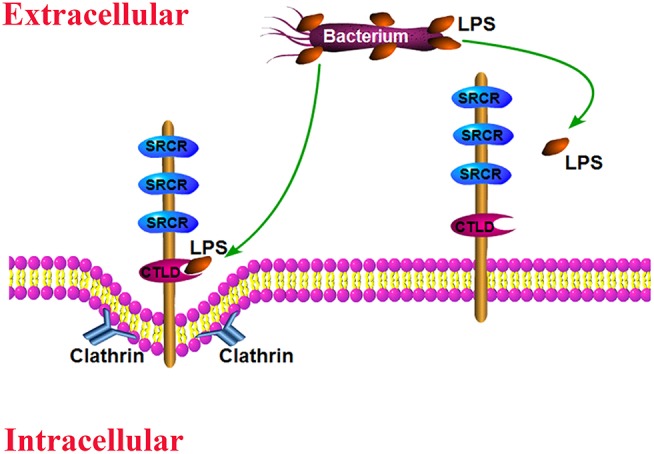
Schematic of the likely antibacterial mode mediated by *Sp*Bark. *Sp*Bark can sense and bind LPS on the surface of Gram-negative bacteria with its CTLD domain, which would induce phagocytosis of bacteria possibly through a clathrin-dependent mechanism.

The invading pathogens in hemolymph are routinely removed by cellular immune responses and humoral immune reactions (3, 41). Hemocyte phagocytosis is an important activity that eliminates diverse invading pathogens. Recent reports revealed that certain receptors or receptor-associated proteins are required to modulate hemocyte phagocytosis activity (30, 39, 42, 43). To further confirm whether *Sp*Bark was involved in bacterial clearance and modulated hemocyte phagocytosis of bacteria, the number of bacteria in cell-free hemolymph and in hemocytes of *Sp*Bark-silenced crabs was counted. Our results showed that knockdown of *Sp*Bark significantly suppressed the clearance of *V. parahemolyticus* in hemolymph and hemocyte phagocytosis of bacteria. These results demonstrated that *Sp*Bark participated in the removal of bacteria in hemolymph and modulated the phagocytosis of *V. parahemolyticus*. Although many immune responses (e.g., the synthesis of AMPs and melanin) are involved in bacterial clearance (41), the fact that the bacterial clearance activity in hemolymph and hemocyte phagocytosis of bacteria were decreased simultaneously by *Sp*Bark knockdown clearly suggested that the bacterial clearance in hemolymph is closely related to hemocyte phagocytosis of bacteria. Knockdown of *Sp*Bark severely affected hemocyte phagocytosis of bacteria, which finally resulted in the decrease of bacterial clearance. *Drosophila* Bark was internalized into cells through clathrin-coated vesicles and then sorted back to the plasma membrane via the recycling endosomes (33). This kind of internalization mechanism might apply to *Sp*Bark. As many scavenger receptors can bind and internalize pathogens, *Sp*Bark may function similar to a scavenger receptor mediating hemocyte phagocytosis through binding and internalizing bacteria. The possible antibacterial mode may be as follows: in hemolymph, the invading bacteria were sensed and bound to by *Sp*Bark CTLD, and this binding behavior would modulate hemocyte phagocytosis, which finally led to the internalization and degradation of invading bacteria (Figure 7). Considering that *Sp*Bark is ubiquitously distributed in major immune tissues, a similar mechanism might occur in the tissue cells of gills and intestine, which might facilitate the removal of external bacteria from environments and intestinal contents.

In this study, *Sp*Bark SCRCDs exhibited much weaker binding activity to LPS than *Sp*Bark CTLD and no binding activity to the other tested polysaccharides, although SRCRD-containing proteins were shown to bind to various ligands participating in immune responses (17, 18, 20, 44, 45). This result suggested that *Sp*Bark SRCRDs might play a major role in other biological processes. Our study revealed that *Sp*Bark SRCRDs possessed binding ability to ac-LDL, implying that *Sp*Bark may be involved in lipoprotein metabolism. *Drosophila* Bark is shown to mediate cell adhesion and functions as an essential component in a core complex of septate junctions (33). Three SRCRDs of *Drosophila* Bark, as the major functional domains, might play a role in this biological process. Given that *Sp*Bark shared similar structural domains with those of *Drosophila* Bark, we speculated that *Sp*Bark exhibited similar biological function through its SRCRDs. Thus, *Sp*Bark might be a multifunctional protein; however, further studies are needed to confirm this speculation.

In summary, we characterized a novel scavenger receptor-like protein, namely, *Sp*Bark, in the present study. *Sp*Bark CTLD possessed much stronger binding activities to ac-LDL and LPS than three SRCRDs serving as the major ligand-binding site. Knockdown of *Sp*Bark significantly suppressed the phagocytosis of bacteria and bacterial clearance in hemolymph. This finding clearly suggested that *Sp*Bark modulated hemocyte phagocytosis of *V. parahemolyticus* through binding to LPS with its CTLD, which was an important activity to eliminate invading Gram-negative bacteria *in vivo*. This study provided new insights into the biological functions of Bark proteins and antibacterial mechanisms of invertebrates.

## Data Availability

The datasets generated for this study can be found in the GeneBank (Accession No. MH595537).

## Author Contributions

X-CL and W-HF conceived and designed the experiments. X-CL, W-HF, and JZ wrote the manuscript. JZ and X-CL conducted most of the experiments. YueW, HM, J-FZ, YuanW, and SZ contributed experimental suggestions and revised the manuscript.

### Conflict of Interest Statement

The authors declare that the research was conducted in the absence of any commercial or financial relationships that could be construed as a potential conflict of interest.
