# Supplementary material for: SpBark Suppresses Bacterial Infection by Mediating Hemocyte Phagocytosis in an Invertebrate Model, Scylla paramamosain
Source: Front Immunol. 2019 Aug 23;10:1992. doi: 10.3389/fimmu.2019.01992 (PMC6716108; doi:10.3389/fimmu.2019.01992)
Supplement: Supplementary file 1 [file Table_1.DOCX]

1 GGTGGTGGTTGTGGTGGTGACAGCATGGCGATGACGCGCATAGTGGTGTTGTGGTGGACACTGGTGGTTGTGACAGCGCGGCCCGTCGTGGCATGTAGTCTTGGA

1 M A M T R I V V L W W T L V V V T A R P V V A C S L G

106 GGAAGCATCGTCAACAGGGTGGAGTGTGACCTGACAGGCTCCCCCTACACAGTGACCCAGGACGTGGTGGTGCAGAAGGGCGCAACTCTCGTCCTCAAGCCTGGA

28 G S I V N R V E C D L T G S P Y T V T Q D V V V Q K G A T L V L K P G

211 GTTACCCTGCAGTTTGATCCTGGTGTGGGAATCACCGTGAAGGGCGTCCTGGAAGCCGAGGGCACGGAGTTGCAGCGGATTACACTGACGAGCAGCCAGCCGCCC

63 V T L Q F D P G V G I T V K G V L E A E G T E L Q R I T L T S S Q P P

316 GCCGCCCCCACGCCCGCGCCTTCTCTGCGCATAGTGGACGGCCCCACCCCCATGCAGGGCCGCCTACAGATCTACTACAAGGACAAGTGGCGCTCCATCTGCACC

98 A A P T P A P S L R I V D G P T P M Q G R L Q I Y Y K D K W R S I C T

421 AATTCCCGCAACTGGACTGCGGCGGACCTGACAGTGGCTTGTCGACAACTGGGATTTAGCGGCGGCAAGTATTGGGAATGGCAGGACCGCGCCAACAACGACACG

133 N S R N W T A A D L T V A C R Q L G F S G G K Y W E W Q D R A N N D T

526 GCGACACTGCTTTACCAGGCGCCGCAATGCTCGGGCCTCGAGGATGACATCACTAAGTGCGCCTGGCAAACACATTCCATGGGCGGCGGCGTCTGCGATATGCAT

168 A T L L Y Q A P Q C S G L E D D I T K C A W Q T H S M G G G V C D M H

631 CCTGACCTCGGCGTGGACTGTGACCCTTACCATGCCCTGTGGCGCGGCACCAACCACTGGAAGGGCATCCTCTTCCAGAACGCGGACTTCGAGGGGCAGCTGTTC

203 P D L G V D C D P Y H A L W R G T N H W K G I L F Q N A D F E G Q L F

736 CACAATACATTATATCAGAAGGTGTCCAAGTCTGTGCTGAGCTTTGTGAACATTGAGTATGCAGGCATGGGGCCACAGCGGGAAGTGTTGTCATCCATCATGACT

238 H N T L Y Q K V S K S V L S F V N I E Y A G M G P Q R E V L S S I M T

841 CGAAAAGTGCCGCCACGCCTGCGTCACGTGACCGTCACGAACAGTGCCTTCAATGGCATCAACATGACCCTCCCCGGGACATTTGTCCATCTGGAGAACGCCGTG

273 R K V P P R L R H V T V T N S A F N G I N M T L P G T F V H L E N A V

946 CTGAAGGAGAACGCCGGTCATGGGCTTTACGTGAATACCTCAACGGGTTCAGTTTTGATCGATCGGGACTCTGTGGTGACAGACAACCAGGCAGATGGAATCAAG

308 L K E N A G H G L Y V N T S T G S V L I D R D S V V T D N Q A D G I K

1051 TATAATTTTCACCATCGCGAGCCAGACAAATCTGCATCAGACACTTTCCAGGACTTTTGTGCTGGGGCATCCAACCTCAACCAAGCTTACCCCATTGTAACAGTG

343 Y N F H H R E P D K S A S D T F Q D F C A G A S N L N Q A Y P I V T V

1156 GCCACACAGGACCGGTACTCCTTCAAGGAATTGAACTGTGAGAAGCTGTTTGTCACTCGGGACAGTGATTTTGTCTTCACTGTACATTTCTCCTACATGCAAGCT

378 A T Q D R Y S F K E L N C E K L F V T R D S D F V F T V H F S Y M Q A

1261 GAAGAGGAGAAGGCAGGCATGGTTGAGGTCAGAGAACAGAATCGGTATGGGAATCTCTTAACCAAGTTTGAATTGAGAAATAACACATTTCCACCTTCTGTTGTG

413 E E E K A G M V E V R E Q N R Y G N L L T K F E L R N N T F P P S V V

1366 TCCCGAGGCAATAGGATCTGGATCAAGGTGACAGCTAAACCTCGTATCCTGTCATTCATCTTTATGGAAGTGATTGCCAGCAAGTACAAGATGTTTGACCTCAGT

448 S R G N R I W I K V T A K P R I L S F I F M E V I A S K Y K M F D L S

1471 GTGCGGGACACCATGGTGCGAAACAACAGTGGGCATGGAGTGGCAGTGGATTACATGAGAAGTCTTGTGCATGTTCATCGTTCCAACCTGACCCATAACTACTAT

483 V R D T M V R N N S G H G V A V D Y M R S L V H V H R S N L T H N Y Y

1576 GGGTCGGGTCTCAATGTACGGCAAGGTGCTGGGGATGTGAATGTGACACACTCCACCATAATGTACAATGTTGGAGATGGTGTCAATGTCACATATGAGGGTGGA

518 G S G L N V R Q G A G D V N V T H S T I M Y N V G D G V N V T Y E G G

1681 GTGCAGAATGTTACCTGGAGTACTGTAGCTGACAACAAGCTACGTGGTGTGGCTGTCTGGTTCAACGAGAGTGGTCAGGATACTGGAATCCATCAAGAAACAGCT

553 V Q N V T W S T V A D N K L R G V A V W F N E S G Q D T G I H Q E T A

1786 GTGGCATATTCTACTTTGACTGGCAACCTGGACGTGGGGCTGCGTGTGGGAAACTTTTGTCGGCCATCTTTCGTAAACATCAGCAGCAACACATTTAGTGATGGC

588 V A Y S T L T G N L D V G L R V G N F C R P S F V N I S S N T F S D G

1891 CATGAAGCTGCTCTTGAGATTGAATCTTGCTGGCTGAGGAGCGATGAGAGGAGAGCAGTGCAAATTGGCCAAAACATGTTTAGGAATAATCACCGTTTGGCCATC

623 H E A A L E I E S C W L R S D E R R A V Q I G Q N M F R N N H R L A I

1996 AAAATATCACCAGCTGTAAATATGAACTTTACTTTGGAATACAATGAATTTCATAATAATAAGCATGGCACTGTCCTTATGTACAATGAAGACAAAGTGGAGCTT

658 K I S P A V N M N F T L E Y N E F H N N K H G T V L M Y N E D K V E L

2101 CCAATGCTCCCATTCAGTGGCATGATACAGAGGAACAACTTCCGAGGCAATAGTGGGTGGTACGTTCTTAGGCTTGGCCTTTCCTTGCTTGGCTTGGACCAGAAG

693 P M L P F S G M I Q R N N F R G N S G W Y V L R L G L S L L G L D Q K

2206 CTGCGGCTAGAGAGGAACGTCATCAAGGAAAATACAGTAGAGGAATTGTTCGTGGCCTTCCACTCCCGCAGCCAAGCATCAGGGGTAGTTTGTATTGGTTCCTCC

728 L R L E R N V I K E N T V E E L F V A F H S R S Q A S G V V C I G S S

2311 AACATTGTCCTTTACAGGAACTTGTTAGATAACCCAGCTTCTAGATATGAGTTGGCCTCCCACACCAGTGATCAGAGCATCCCCATCCAAGCTACACACAACTGG

763 N I V L Y R N L L D N P A S R Y E L A S H T S D Q S I P I Q A T H N W

2416 CTTGGTAGCAAGTTTGAGCAGGAGATCTTCAGGAGGATCCTTGACCGTCGAGACCTATACAATTTGGCGCTTATACATTTTCATCCTTACCTGCTGAGCAATAAC

798 L G S K F E Q E I F R R I L D R R D L Y N L A L I H F H P Y L L S N N

2521 AATGTGGAAACACCTGTCATGCAGGAGGGCCCTGAGAGTGAACCAGAATTCTTTGACCCAGCAGACAACCACATTATTGGAGGAGAGGTGAACGGTGAGGTGACA

833 N V E T P V M Q E G P E S E P E F F D P A D N H I I G G E V N G E V T

2626 CTACGTGATGGCCTCTATACTGTCACACGAGACATTTACGTTCACAAGACAGGCACGCTGACTATTGCCTTTGGCACTACACTTGAGTTTGCTGAAGGCATGGGC

868 L R D G L Y T V T R D I Y V H K T G T L T I A F G T T L E F A E G M G

2731 ATGATGGTGGCTGGGCTGTTGCGTACGGAAGGCTCTGGAACTCGTGATGTTAAGTTCACTCTCCATGGCACTACTGAGCAGGAAGAGGCACTGGAGAGGGCTGCA

903 M M V A G L L R T E G S G T R D V K F T L H G T T E Q E E A L E R A A

2836 TATTTCTCTGGGGAAGAAGAAGCCATACCTCTCAATGCCACAACCAATATGACTCTAACTGAAGTGCACAACCTCAATGTGACACAGGAGGAGGACATGACCCCT

938 Y F S G E E E A I P L N A T T N M T L T E V H N L N V T Q E E D M T P

2941 TCTGTGCTTGTTAAGCTAGTGGGTGGCAGAAACAGCCATGAAGGCAGACTAATGGTTTGGGTTGATGGCCAGTGGGGAACTGTGTGCAACCATGGCTGGAACCAG

973 S V L V K L V G G R N S H E G R L M V W V D G Q W G T V C N H G W N Q

3046 GATGCAGCAGCCATAGCTTGCCACCAGATGGGACTGGTGCTGAACCCTGAGGACTGGGATCTGCAACCCAGCCAGCTGCCATCTGAAGGCCAGACCAGCCCCATT

1008 D A A A I A C H Q M G L V L N P E D W D L Q P S Q L P S E G Q T S P I

3151 CTTATAAGCAATGTCCGGTGTGACGAGTTTGACACTGACCTCACAAAGTGTATCAGTGATCGGTACTGGGAGTTAGAGAATTCGTGTTCCCATGCTGAGGATGTG

1043 L I S N V R C D E F D T D L T K C I S D R Y W E L E N S C S H A E D V

3256 GGCATCCGTTGCTACCCTGGCTCCTGGGCTGGCATCCGCTTGGGCATGACAGCACATGAAAGCCACATGAAGGGCGTGATCATAGAGAAGGCTGGTCTCTTTGAT

1078 G I R C Y P G S W A G I R L G M T A H E S H M K G V I I E K A G L F D

3361 TACACTACCCGCACTTTCAAGCCAGCCCTTCAAATTGACTTCCACCACCACGTCATCCAGGATGTGGAGATCCGTAACAACCTGCAGGACGGAGTGGGTGTGGTG

1113 Y T T R T F K P A L Q I D F H H H V I Q D V E I R N N L Q D G V G V V

3466 TACAGTGACCAGTATGCCATTGTAAACCCCAGTGCCAGAGTATTCCACTCCTGCACCTTCTCTGGGAACAGACGGCATGGTATCACCCTCAAGCAGCTGGGTGTA

1148 Y S D Q Y A I V N P S A R V F H S C T F S G N R R H G I T L K Q L G V

3571 AACATTACTAACTCCAATTTGGAGCGTAATGATGGTTCTGGCCTGCACTTCAATCCTTTCTTGAAGAGAGCAGAGCAGAGGGAACTGGCTAGCTGGCTCAAGCTG

1183 N I T N S N L E R N D G S G L H F N P F L K R A E Q R E L A S W L K L

3676 CAGCAGGATCAATATTACAAAATACCAGAGGGCCCAAAGAATGTGCGGCTAGAAACCAACCTGCCTAAGTACTTCATCACCCAGACCTTGCGTGGTGCTGGTGCC

1218 Q Q D Q Y Y K I P E G P K N V R L E T N L P K Y F I T Q T L R G A G A

3781 AGGGCCACCATCACAGTTTCGACGTCACATTCTAACGTGATTGGCATCCAGGTGCTGAACCCCATCATTCCTGAGAGCACTGAGCAACTGGTGATTTATGACTAC

1253 R A T I T V S T S H S N V I G I Q V L N P I I P E S T E Q L V I Y D Y

3886 CAGGAGATCATTGAAAGTGAGGACATTGATCATTGGGACTTGCGAGGGGACCAGGTGGCCTTCCCTACCACCTCTAGTAGTTACGCCATCACTATTGACTACAGC

1288 Q E I I E S E D I D H W D L R G D Q V A F P T T S S S Y A I T I D Y S

3991 TCAGGACCTTATGCACTCGGAGATGTCATCATCCTCCTAACTGCCATTGACAGGCGAGACATTGCACAAGCAGGAGACCTCAGGAATTTCCGAGCCAAGTGGCCA

1323 S G P Y A L G D V I I L L T A I D R R D I A Q A G D L R N F R A K W P

4096 ATGACATACATTGACCAGACTCGCATTCAAAACAATGACATTGGCATCTCAACTCTCCACTATAACAGGTATCTCACAGATGATGAGGACCATCTGCTACGGTGT

1358 M T Y I D Q T R I Q N N D I G I S T L H Y N R Y L T D D E D H L L R C

4201 GCCAATGAGAGCATTGTTGTGGTGGGCTCCCATATCAACAACAACCAGAACCAAGCTGTCTACACCCTCTCACCTTTCCGTGTGCGCATGGACAACGACGACATT

1393 A N E S I V V V G S H I N N N Q N Q A V Y T L S P F R V R M D N D D I

4306 GCTGAGATCACTTTCATGTTCAATAGCACCACAATTTCTGGCAATGGGCGTGGCATTGATCAGTACAGCTGGGATGTGCGGGAGTCTAACAACCTGTTCCATTGG

1428 A E I T F M F N S T T I S G N G R G I D Q Y S W D V R E S N N L F H W

4411 GTGCTGGATGAGGTAGTGATGGAGAACAATGGTGGAGGAGGGATAGTACTGTCACTTCCCTATGTCTGGCAGTACACAGAGAACTTCACCCACACACTTTACATC

1463 V L D E V V M E N N G G G G I V L S L P Y V W Q Y T E N F T H T L Y I

4516 AATTCTTCATCCTTCATCAATAATGCCCAATTCCAGTTTGTAGTGGATGGCCATTTTGCACGCTTCAACATGACATACTCAAGGTTTGAAAATAATGTCTGTCAG

1498 N S S S F I N N A Q F Q F V V D G H F A R F N M T Y S R F E N N V C Q

4621 CGAGGATTGTTGTCAGTACAGGGTATGGAGAAGGAAATGTTCCTTCATGACAACCGCATTTCCAGTAATGTGGGTTCCTTTATGGTTGAGTTCAACACTAACAGC

1533 R G L L S V Q G M E K E M F L H D N R I S S N V G S F M V E F N T N S

4726 CAGAGTGGAATCTTGGGCTCAGTCAGTGCTTACTTTGAGTACAACCTGGTACAGAACAACAAACGCATGCCACAGACATTCAGTTCTGTATCACGGGCTCATCAG

1568 Q S G I L G S V S A Y F E Y N L V Q N N K R M P Q T F S S V S R A H Q

4831 CCTGCAAGCTATACCTTGGCTGTGCGAGGTCTCCAGAAAGTGAACATCACCCACAACCTCTTGGGTTACAATGAAATGGACTATGAGCTGCTGGCAGGCCTCTAC

1603 P A S Y T L A V R G L Q K V N I T H N L L G Y N E M D Y E L L A G L Y

4936 ACCTCTCGCTTGAACAATTACCTCAATGTAGAGGCAAACTGGTGGGGCACTCGCAAGGCAAAGGAGATTGAACAACTCATTTTTGACTTTGATGACTGGAATAAC

1638 T S R L N N Y L N V E A N W W G T R K A K E I E Q L I F D F D D W N N

5041 TTTGCCCTGGCAGACTACCTTCCTTACCTCATAGAGGACAACATCGAGGCTCCTGTATCCTCTGTTGGATCCTATGCTACTCAGGACACCACAGACATCAATTCC

1673 F A L A D Y L P Y L I E D N I E A P V S S V G S Y A T Q D T T D I N S

5146 CTTGGAGGCCGTTTGCTGCATGACTTGCGTTTACCAAAGCGAGATGAGCCGTATGTGGTGAAGTCTGACTTGACAGTTATGCCGGGAGTGACACTGGTGGTGGAG

1708 L G G R L L H D L R L P K R D E P Y V V K S D L T V M P G V T L V V E

5251 GCAGGTGCTACTCTTGAGTTTTTACCAAGTGTAGGACTACTGGTGTTGGGTCGCTTGGAGGCAATTGGCACCAAGGACCAGCGCATCAAAATGCAGCCTGTACAG

1743 A G A T L E F L P S V G L L V L G R L E A I G T K D Q R I K M Q P V Q

5356 CCTTCTGTACCCTTCCAGCACAGAACCACCCGCCATGCTAAGACCTCACTGGAAGCTGTGAGACTGTGTGTGGAAGGGGAGTGTGGTGGCCGAAGAGAAGGGTTC

1778 P S V P F Q H R T T R H A K T S L E A V R L C V E G E C G G R R E G F

5461 CTGGACATCTACAATGGCACTACCAAGCAGTGGGTGCCCATCTGTGATGACCGCTTCACTGAGCGCAACACTGAAGTGGTGTGTAGACAGCTTGGCTACAACACC

1813 L D I Y N G T T K Q W V P I C D D R F T E R N T E V V C R Q L G Y N T

5566 ATTCACACCTTCCATGACCGCAATAAGAGGATAGAGATGTTCCCCAATGCCCTTATTCGCATTCATTCCTGGCCTGATCCCATAGAGTGTGATGGCACAGAAGCA

1848 I H T F H D R N K R I E M F P N A L I R I H S W P D P I E C D G T E A

5671 GCGCTGAATCAGTGTCCCCTCAGGATGAATGGCCAAATCTACGATCACACCTATAGTTGTGCTTGGAATGGAGACTTTGTGTACATCTCTTGTGGAGACCTGAAT

1883 A L N Q C P L R M N G Q I Y D H T Y S C A W N G D F V Y I S C G D L N

5776 CTTGAGGACACCAAGCTGGAGTACTGGGGTGGTATCAGGTTCTCCATCCCCAACTTTGAACACATGGATGTATACAGTCGCATCCATGATCCCAATGCCCCTCAC

1918 L E D T K L E Y W G G I R F S I P N F E H M D V Y S R I H D P N A P H

5881 CATGCCCATGCCCACCCTATGGTGCATGGAGAACATCAAACTCCAGTCTACCCTTCCATTTTGGAATGGGTTGAGGTGGTGGGAGCTGGCATCCTTCATAATGAC

1953 H A H A H P M V H G E H Q T P V Y P S I L E W V E V V G A G I L H N D

5986 AAGTCTCCGGCCATCATGTCCTTCCATGAAACACCTAAACTGCGTGGCGTCACCATCAAGGACTCAGCCTATGATGGTCTTACTCTTATTTCAGCCACATATGGC

1988 K S P A I M S F H E T P K L R G V T I K D S A Y D G L T L I S A T Y G

6091 TTTGAGATGCTCTATAACAAATTTGAGAACAATCTTGGAGTGGGAGTGACACTGATTGGACTGACAGGAGAGACAAGAGAGACAGAAGAATCATCTTTCTTCCCT

2023 F E M L Y N K F E N N L G V G V T L I G L T G E T R E T E E S S F F P

6196 CTGACCAATGTACGGCTCCCTTATCACACCTTTGGGATGGTGGACATGTGTGACTCAACCAAGGAGCTGCGAATTGAGGAGCGTATCTTGCTCTACTACAAGTAT

2058 L T N V R L P Y H T F G M V D M C D S T K E L R I E E R I L L Y Y K Y

6301 GACAATAGACCCGTTGACTGCATCAAGATCTTCTCTTCTGTGTTCAATGTGAAGAACTTTGGATTTAGGCTACTCCATCTCAATCTGTATGATTCCACCTTCAAT

2093 D N R P V D C I K I F S S V F N V K N F G F R L L H L N L Y D S T F N

6406 GGGGAGCCACTGGTGCGGGACCGGCTGGTGTTGTATGATGGGGACATCTATAACTACACTACCCAAGTTTTTGCTGAGGTGCATGCTCGCTCAGATAACCACATG

2128 G E P L V R D R L V L Y D G D I Y N Y T T Q V F A E V H A R S D N H M

6511 CGTTTCTTCAAGACTGCTGGCACCAGCCTCTCAGTGGAGCTTCATGCCACAGGAGCCTCAGGAGAGCTTGGCTTTGTGGCTGAAGTGGTGACTCTGCCCATCTCT

2163 R F F K T A G T S L S V E L H A T G A S G E L G F V A E V V T L P I S

6616 AACCTAGGAATCAACCGTAACATCCTACACAACTTCACCTACAACGAGTACCACAACAATGTGGAGGGAGCCATATTTGCTGCCACTGCTGGTGAGGTAAATCCA

2198 N L G I N R N I L H N F T Y N E Y H N N V E G A I F A A T A G E V N P

6721 TGGCTCTGCTTCTCTTATAGTCGCATAGAGAACAATGGACGGCAGTTGTATGGCAACTTCACCACAACACAAGCTGCTGTACGCCTTGATGTGCAAAACATGCAA

2233 W L C F S Y S R I E N N G R Q L Y G N F T T T Q A A V R L D V Q N M Q

6826 GATGTCTATTTTAAGAACAATCTGGTGCGTAACAACACTGGCGGGCTGCACATCATTGCTGGCTCCATTGGTGCAGCCACCAAGCTACAGGCCAATGTGACCAAC

2268 D V Y F K N N L V R N N T G G L H I I A G S I G A A T K L Q A N V T N

6931 AACTTGTTTGAGGAAACTCAACACTGGCAAGCTCTCTACATGGCCTCCAGGGAAAATTCTGCCTACCAGCATGCCCTTATAGCCTACAATGACTTCAGTTGGTCC

2303 N L F E E T Q H W Q A L Y M A S R E N S A Y Q H A L I A Y N D F S W S

7036 TTCTCGCCCTACCATGATGTCATCACTCTGGCTCAGGTGGTGTCTGTCTTCACTCACAACTATGCGCACAGCAACATTGGCCGCCACATTCTGGACATCTATGGC

2338 F S P Y H D V I T L A Q V V S V F T H N Y A H S N I G R H I L D I Y G

7141 TTCCAAAAAGTCCGTCTCCCTGTATACCAGACCACCTCACACAACAGTCTTGCAAAGAATCAGGCATGGGATCCCACCTACCAAGGCACAGTTATTGCCGGGAGT

2373 F Q K V R L P V Y Q T T S H N S L A K N Q A W D P T Y Q G T V I A G S

7246 GCAGGGCAACAGTTTGTGGACAATGTTTTCTATAACTGGGACAACGCATATGAGTTGGTGGCTGTGAACGAGTCTGTGCGGTGCAACATTTCCACCGAGTGCACT

2408 A G Q Q F V D N V F Y N W D N A Y E L V A V N E S V R C N I S T E C T

7351 GACTCTGAGAGCTTCACTTTGGAGAAAGTAGTAGTCACTGACAGGTCTGATGTATGGAAGACTCCCATTGATGCACGAAACAACTGGTGGGGCTTCAATACTTCT

2443 D S E S F T L E K V V V T D R S D V W K T P I D A R N N W W G F N T S

7456 GTGGCAGTGTCAGGCCGTATCCATGACAAGACTGATGATGAGACCCTCCTACATGTCGATTACAGTAACTGGAAGCTAAACAACTACTCACTGCTGCAAGGATGT

2478 V A V S G R I H D K T D D E T L L H V D Y S N W K L N N Y S L L Q G C

7561 GAACCAGGCTACACCTTAATAGGGGACTCTTGCTACTTGTATATAGGGGCACCTGTCACTTATCATGAAGCTAAAGAATTCTGCAGGAAAGACAATGCTTCACTG

2513 E P G Y T L I G D S C Y L Y I G A P V T Y H E A K E F C R K D N A S L

7666 CCATTCCTTCAGAAGTGGTATTGGGAAGTCCAACATTGGTTGTTAGAGCAGCAACCAGAATACCTATGGGAGTCAGACATAGTGTGGGTGCAGCACCTGGACGTC

2548 P F L Q K W Y W E V Q H W L L E Q Q P E Y L W E S D I V W V Q H L D V

7771 ATCAGTGGCTGTGCTGGGTTTGTCTACCGTCAGGTGCGCTCTGTTGACTGCAACCTGAAGCTGCCATTTATCTGCGAGTCTGATGCAGATGAAAAGATTGACCAG

2583 I S G C A G F V Y R Q V R S V D C N L K L P F I C E S D A D E K I D Q

7876 CTGGCCTGGGCTTCAGACCCTCTGGCAGTGGCAGCCATTGCTATGACTATGGGTTGCCTCATCCTGGTGGTTGCCTGCTTGTCTTGCTGGGTTTGCAAGTCTCGT

2618 L A W A S D P L A V A A I A M T M G C L I L V V A C L S C W V C K S R

7981 GAACGTCGCAAAGAAAGAATCATGCGCCGCAACTCAATCCGTGCCTCCATCCGCTCCAACAGATCAGCCATTTCCACCTCCACCACCACAGGGGGCTTCAGCGAT

2653 E R R K E R I M R R N S I R A S I R S N R S A I S T S T T T G G F S D

8086 CTCACACGCCGCAGGATAATTGAGGACCCTCAGCATGCAGTGCCAATGAAGGGGCAGGAAGCTGTTGGGGGTGCTGCCAGGATGAATGGACTCCATGGCTCCTTT

2688 L T R R R I I E D P Q H A V P M K G Q E A V G G A A R M N G L H G S F

8191 GACTCTATTGCCAAGTCTGGGCTTAATTCTTCCATAGATGAAGATCCAAGCTTTGTTGTATATGAGGAGACCACTCCCTCAATTCCTCCTGGTTACTCTTCTCAG

2723 D S I A K S G L N S S I D E D P S F V V Y E E T T P S I P P G Y S S Q

8296 CTGGATGACAGGTTCTCCGGTGATCCCCAGCTTGAGAATCACAATGTGAACGAACTCCTTCGGCCAAGCTTCAACATAACCTTCCAGAACCAGGGCTTCAGGGAT

2758 L D D R F S G D P Q L E N H N V N E L L R P S F N I T F Q N Q G F R D

8401 AACTCAGCATTCTCCTCTCGTGAAAACTCCAGCTTCATGCCCCAGACTACTTCTGAGGCCTGGGTGACTGATGATCAACCAGCAAGTTTTGGTCAACAGTCTTTT

2793 N S A F S S R E N S S F M P Q T T S E A W V T D D Q P A S F G Q Q S F

8506 ACTACCTATGGGCATGACTCCCGCCTGCCCCCTCTCCAGCCGCAGCTGTCCCCAAGGCTCAGGCTTTTGCCTCCGCCACCCTCCAGAAGGGAGTTTCCAAGTCCA

2828 T T Y G H D S R L P P L Q P Q L S P R L R L L P P P P S R R E F P S P

8611 AGGTAGGCAGACCAGGGTAGTGAGGCACTGAACATGGTTGTCTCCTCATGTGATTTTGAATCAGGAAATACTCTGATGCATGACAAATTACTAATTTGCATGAAA

2863 R *

8716 TAATTTTCAGTTTGTGATGTGATTTAAGAAAATTATAATGCACATTTAGTAATGCAAAACTGCATCAGGTCTCTTTATAATGCATGACAAATTACAATGCCTGAA

8821 TCTAACAAAATAGTACTCCAAGGTTATTTTATATGTAATTGATCCTAAGTATTGTTATAATAATGTATGCATGGAGAATGCCCTTTCCTTCAGCTTTGAGAACTG

8926 TAACTCAATAAACAAAAACAGTTTGTTGGTAAAATATTCATACCAGCATCCATTCAGTCTCTTCTGTTGTCCATCATAGCATGCAGTAAATTCCTAACCAACAGT

9031 AGTCCTATGCTTGAGTAGGAATATTAGAAACAGTCTACAGTTCAGCAGGTATTAGTATGGTAATGGTATTGTGATATGCAGGAAATTAAACAAATCATTCTGACA

9136 CTTCCAGCTACCAGCAAAGTAGCAGCTCACCTCAGATGCCTCCCAACAACCAAGCCTGGCCTTTCAATGGTGGTGCCTCCAGCCTCCAACCAGAGGAATTGTCAA

9241 TACATGGCTCCACAGCACAACTGGCTGGGAGTAGCATAACTACGGACTCCACCTTAGAGATGAAAAAAGAGGCTGAGGACCCTCACCTGTCTGCCACACTTCCCC

9346 GGGAACAGGGTCCCACCTACCAATACATGTCTGCCAATGTTCTCAGTGGCCAAAACTCAGGCTCAAGCTCAGATAATTCTGCCCATGGATATTTCTCTGGACGGT

9451 CCCAAAGTCAACCTCTGGAGACAGCTATGTGAGATTATAGTTCTACAAGTGATGTTGGCTTGACAATAATTGTAGAGAGCATGATCTCAAAGGAGGGTATCATAA

9556 ACCAATATAGTGAACAGCAGTGACCATCCAGCACATATACTCATTACTTGAAGTGTTAGTTGCACATGAAAATGCAACATGAAACACATGGTGAGGACATTGTTC

9661 CACCACATGAGACCATCTACTGAATGTGGTGACCAGGTGAGATAATTTATAAAATTATGAGTTTTCTACTTCCACATTGCTAGTGGTAATTAGAGCTTCATTACA

9766 TTTGGCTTTATAGAGCTGAAGCAAAGAAATATTTATCCATTGCAGTGGAATTTACAG**AATAAA**AGTAGATTACAAGAACTAGGTTAGCACAAGAGAATTTAATGT

9871 TCTTGAGCAGTTTATGAGCATATCAGTGAGGCCTACTGAGTGCATTTAAATAGCATTTACAGTGTCATTATCACCAGACAAATACAATGAAATTATAATTCTCCT

9976 GACATTTTTGTAATAATAACATTCTTCACCAGATATTATATTAGAAAGCGTTTACTTCATAAAAAAAAAAAAAAAAAA

**Fig. 1S.** The full-length cDNA and translated amino sequences of *SpBark*. (A) The signal peptide is shown in red, and the stop codon is indicated by an asterisk (*). Three SRCRDs and a CTLD are shadowed. The transmembrane domain was boxed. The classical polyadenylation signal is bold and highlighted.
